# Physical Activity and Its Correlates among Adults in Malaysia: A Cross-Sectional Descriptive Study

**DOI:** 10.1371/journal.pone.0157730

**Published:** 2016-06-22

**Authors:** Tam Cai Lian, Gregory Bonn, Yeoh Si Han, Yap Chin Choo, Wong Chee Piau

**Affiliations:** 1 Department of Psychology, Jeffrey Cheah School of Medicine and Health Sciences, Monash University Malaysia, Bandar Sunway, Malaysia; 2 Japan Society for the Promotion of Science, Tokyo, Japan; 3 Department of Education and Human Development, Nagoya University, Nagoya, Japan; 4 Department of Psychology, UCSI University, Kuala Lumpur, Malaysia; 5 Department of Medicine, Jeffrey Cheah of Medicine and Health Sciences, Monash University Malaysia, Bandar Sunway, Malaysia; IRCCS E. Medea, ITALY

## Abstract

Obesity and rates of non-communicable diseases linked to physical inactivity have increased dramatically in Malaysia over the past 20 years. Malaysia has also been identified as one of the least physically active countries in the world with over 60% of adults being essentially sedentary. This study examines the relationship of socio-demographic factors to physical activity among 770 adults from 3 Malaysian states. Physical activity levels were significantly related to ethnicity, gender, age, occupation and educational level. Controlling for inter-relationships among these variables; age, gender, Chinese ethnicity and education level were found to have unique effects on total physical activity, as well as moderate and vigorous exercise. As would be expected, younger people were more physically active, engaging more in both moderate and vigorous types of exercise and males were generally more active than females. Contrary to findings from many developed countries, however, more educated Malaysians were less likely to engage in all types of physical activity. Ethnic Chinese participants, and to a lesser degree Indians also consistently reported lower levels of activity. Possible intervention strategies are discussed that specifically target ethnic and cultural norms related to physical activity. Future research programs exploring barriers to participation and perceptions of physical activity, as well as programs to encourage active life styles among youths are also suggested.

## Background

This study looks at physical activity and its correlates among the Malaysian population as a way of better understanding, and hopefully addressing, the rapid increases in obesity and non-communicable diseases that are currently plaguing the country. According to the National Health and Morbidity Survey, over the previous 30 years, rates of physical activity among the Malaysian population have decreased dramatically, while rates of type-2 diabetes have risen 4-fold [1,2], and obesity has risen by 280% [3]. The World Health Organization [4] as well as the Malaysian Dietary Guidelines [5] recommend that people engage in at least 150 minutes per week of moderate physical activity, lower levels of activity being close correlates of obesity and non-communicable diseases such as type-2 diabetes [6,7].

Generally, it is recognized that changes in diet and physical activity are a natural result of economic development. High-calorie foods tend to become more easily available and mechanization reduces the amount of physical exertion involved in many daily activities [8]. Such factors, however, seem to be especially pronounced in Malaysia, with many indicators showing Malaysia to be among the most sedentary nations in the world [9,10]. To date, most research on the correlates of physical activity has focused on high-income, western countries [11]. Thus, it is not well known, at this point, what patterns of physical activity exist in Malaysia as well as other middle-income, developing countries.

This paper consists of an in-depth exploration and extended analyses of data collected for a previous study related to the spread of type-2 diabetes among Malaysians [12]. The previous study found that, overall, levels of physical activity were lower for those with higher educational levels and greater feelings of internal control. The current paper, thus, focuses solely on physical activity as an area of interest. It provides much more in-depth analyses and interpretations of the correlates of activity as well as its relationship to various demographic variables. Because Malaysia is a multicultural nation with large portions of the population identifying variously as Malay, Chinese, and Indian, we felt it important to better understand how such cultural factors relate to physical activity, or the lack thereof.

### Previous Research

This section reviews findings from Malaysia as well other nations regarding the relationship of demographic variables such as ethnicity, education, and gender to physical activity.

#### Gender

Women in predominantly Muslim countries such as Malaysia seem to be at a particularly high risk of being physically inactive [13, 14], likely due to the fact that many Muslims view highly physical activities as inappropriate for women. Men tend to be more active, in non-Muslim countries as well [15, 16, 17, 18, 19]. But this trend appears to be especially pronounced throughout the Muslim world.

#### Ethnicity

Evidence from Malaysia also suggests ethnic differences in physical activity levels. Teh et al. [20], for example, found “Other” (usually indigenous or mixed) races to be most active physically, and Chinese the least active, with Malay and Indian participants falling in between. Similarly, Singaporean Chinese engaged in significantly lower levels of physical activity compared to their Malay and Indian peers [21]. Elderly Malaysian Chinese, however, were found in one study to be more active than their Indian and Malay counterparts [22].

#### Education level, occupation type and marital status

Findings from developed countries generally associate higher educational levels with increased physical activity [16, 17, 19]. Also, less educated individuals tend to be less active when not working, while highly educated people tend to be more active, suggesting different attitudes towards physical activity among more and less educated individuals in developed countries. Perhaps, for example, less educated individuals associate physical activity with work, while more educated individuals tend to associate physical activity with leisure or healthy lifestyles.

Studies of occupation and activity also suggest a relationship between socioeconomic status and exercise in western, developed nations. In a large scale study, Burton and Turrell [23], for example found that blue-collar workers were about 50% more likely to be insufficiently active compared to professional and white-collar workers. Similarly, a longitudinal study of workers aged 40 to 60 years old found that, over a period of 5–7 years, physical activity during leisure time increased among professional and white-collar workers while decreasing among blue-collar workers [24].

Other results from western, developed countries suggest that married individuals tend to be more active. Engbergm [25], for example, found that divorce/separation related to decreased physical activity. Pettee [26] found that married older adults, both male and female, were more active than their single counterparts. A ten-year longitudinal study by King [27] also found that transitioning from a single to married state led to increased physical activity.

#### Age

Generally, physical activity tends to decrease with age. Hawkins [16], for example, found this to be true for all ethnicities and races in the USA. A study of 19,145 Malaysians [20] produced similar results.

### Objectives

This study aimed to create a descriptive portrait of patterns of physical activity among various groups within Malaysian society. We hope that a better understanding of the nature and distribution of current risks will help improve the focus and effectiveness of future interventions and policy initiatives. Based on previous findings our general research questions were: How do gender, marital status, educational level, occupational types and age relate to physical activity in Malaysia? And, are these relationships influenced by an individual’s ethnocultural identification? This information, we felt, would be a good starting point for guiding future research and intervention programs aimed at addressing the specific needs of various groups within this diverse population.

## Method

### Sampling

In order to conduct comparisons between ethnic groups, we set an initial goal of 800 participants with an approximate ethnic distribution of a 40-40-20 (Malay, Chinese and Indian) ratio for ethnic groups. This quota sampling approach was deemed to be in line with theoretical generalizability rules proposed by Calder [28].

Data was collected from three Malaysian states: Selangor, Penang, and Terengganu. Surveys were distributed on weekend days at shopping areas that are frequented by people from most levels of Malaysian society. Data collection occurred from December 2013 to January 2014. Participants were paid RM20 (about $5 US) to participate in the study. Surveys were collected from a total of 794 people. From that initial sample 24 participants were excluded for incomplete responses or other disqualifying circumstances. Thus, our final sample included 770 participants. All were over 18 years old: Ages ranged from from 18 to 77 years (M = 30.69, SD = 13.03). There were 308 (40%) males and 462 (60%) females. All participants were Malaysian residents; 34.5% were ethnic Malays, 40.9% were ethnic Chinese, 19.4% were ethnic Indians, and 3.8% were of other, or undefined, ethnicity. Participants were fairly educated: 50.1% had attended high school and 41.8% had some post-secondary education (see Table 1) This same data set was also analysed in Tam et al. [12].

**Table 1:** Descriptive Statistics of Participants (N = 770) (Reproduced, with permission, from [12]- Tam et al., 2014.).

Variable	Frequency (Percentage)	Mean Age
Gender		
Male	308 (40%)	32.38
Female	462 (60%)	29.56
Nationality		
Malaysian	728 (94.5%)	30.76
Non-Malaysian	40 (5.2%)	29.88
Ethnicity		
Malay	266 (34.5%)	27.28
Chinese	315 (40.9%)	29.65
Indian	149 (19.4%)	39.69
Others	29 (3.8%)	26.55
Marital Status		
Single	490 (63.6%)	24.49
Married	249 (32.3%)	41.81
Divorced	14 (1.8%)	41.86
Widow/widower	13 (1.7%)	42.38
Others	2 (0.3%)	21.00
Occupation		
Housewife	56 (7.4%)	45.38
Student	185 (24.0%)	20.59
Business Owner	58 (7.5%)	42.00
Professional	44 (5.7%)	34.32
Administrator	20 (2.6%)	36.80
Sales/service	280 (36.4%)	29.20
Others	116 (15.2%)	35.74
Education		
Primary School	45 (5.8%)	40.73
High school	386 (50.1%)	32.31
Pre-university	137 (17.8%)	29.25
University Degree & above	185 (24.0%)	26.06
Others	11 (1.4%)	33.00
Family History of Diabetes		
Yes	242 (31.4%)	30.39
No	526 (68.3%)	31.27

### Data collection

Questionnaires were distributed to groups of 3–5 participants at a time to ensure proper supervision of the data collection process. A central investigator was present at all times during data collection. Ethics approval was obtained from Human Ethics Team of Australia (Monash University Human Research Ethics Committee (MUHREC: CF12/3382-2012001623). All research assistants were trained in proper techniques for recruiting participants, administration of questionnaires and screening of data.

### Measures

The entire questionnaire was translated from English to Malay (Bahasa Melayu) and Mandarin (Chinese) using standard back translation procedures. Our translated versions of these scales were validated in an earlier pilot study [29].

#### Multidimensional Health Locus of Control Scale; MHLC [30]

The MHLC assesses perceived level of personal control, over health-related behaviour. It is an 18-item instrument that measures three dimensions of locus of control: Internality of health locus of control; powerful-other locus of control; and chance locus of control. All 18 items are arranged on a 6-point Likert scale ranging from “strongly agree” to “strongly disagree”. Higher scores indicate stronger belief in the dimensions of locus of control. In previous studies the internal reliability (Cronbach’s α) for this scale ranges from 0.67 to 0.77 for all three dimensions. This scale also has good criterion validity, correlating with participants’ state of health.

#### Simple Lifestyle Indicator Questionnaire; SLIQ [31]

The SLIQ was used to assess the lifestyle of the participants. There are five domains that are assessed which are diet (3 items), activity (3 items), stress (1 item), smoking (2 items) and alcohol consumption (3 items). This is a 12-item instrument. All the items excluding the items in the alcohol domains are arranged on a Likert scale. For each component, a raw score and a category score can be calculated. To provide equal weighting for each component, the overall SLIQ score is based on the 5 category scores. Each component has a category score of 0, 1, or 2, so overall SLIQ scores can range from 0 to 10. Higher scores indicate more healthy lifestyles. In this study, raw scores were calculated for physical activity, ranging from 0 (< 1 time per week) to 3 (≥8 times per week). The test-retest reliability of the 12-item scale has ranged from .63 to .97 in previous studies. Cronbach alpha was .58 for the diet domain and 0.6 for the activity domain. There was a correlation coefficient of .77 between the scores of the participants and blinded raters.

#### Demographic information

Participant’s gender, age, ethnicity, educational level, occupation, family medical history and general health management method was gathered. No personally identifiable information (e.g., name, IC number) was collected to ensure the confidentiality of the participants and their responses. Participants have given contact information to the researcher if they wish to be informed of the research results.

## Results

### Physical Activity Level

A descriptive analysis was conducted to examine the mean, standard deviation, and frequency of different levels of physical activity (i.e., light, moderate, and vigorous). The results showed that more than 40% of the participants engaged in physical activity from 1 to 3 times per week, specifically for light exercise (50.4%), moderate exercise (42.9%), and vigorous exercise (44.9%). On the other hand, a total of 9.9%, 16.3%, and 28.1% of participants did not engage in any light exercise, moderate exercise, and vigorous exercise respectively. A total of 25.1%, 27.7%, and 15.1% of participants who engaged in light exercise, moderate exercise, and vigorous exercise respectively exercised for 4 to 7 times per week, whereas a number of 14.6% (light exercise), 13.1% (moderate exercise), and 11.8% (vigorous exercise) of participants engaged in exercise 8 or more times per week.

#### Ethnic differences in physical activity level

One-way ANOVA showed that there is a significant difference between ethnics in terms of their overall physical activity level, F (3, 755) = 4.75, *p* <.01 (See Table 2). A Tukey post-hoc test revealed that, Chinese (*M* = 11.51, *SD* = 5.86) engaged significantly less physical activity compared to Malay (*M* = 12.80, *SD* = 5.77) and other races (*M* = 15.14, *SD* = 5.22). When further analyzed into the different levels of physical activity, one-way ANOVA showed significant differences among ethnicity in terms of light exercise, F (3, 753) = 4.65, *p* <.01, moderate activity, F (3, 754) = 2.68, *p* <.05 and vigorous activity, F (3, 753) = 6.26, *p* <.01 (refer Table 2). Tukey post-hoc tests revealed that Chinese (*M* = 11.51, *SD* = 5.86) engaged significantly less light exercises compared to Malay (*M* = 15.14, *SD* = 5.22) and Indian (*M* = 12.80, *SD* = 5.77), whereas other races (*M* = 1.79, *SD* = 1.18) engaged more in vigorous activity compared to Malay (*M* = 1.18, *SD* = .91), Chinese (*M* = 1.02, *SD* = .91) and Indian (*M* = 1.02, *SD* = .98).

**Table 2:** One-Way Analysis of Variance of Physical Activity by Ethnicity.

Variables	Source	*df*	*SS*	*MS*	*F*	*p*
Overall Physical Activity	Between groups	3	493.62	164.54	4.75	.00
	Within groups	755	26149.75	34.64		
	Total	758				
Light Activity Level	Between groups	3	10.16	3.39	4.65	.00
	Within groups	753	548.92	.729		
	Total	756				
Moderate Activity Level	Between groups	3	6.61	2.20	2.68	.04
	Within groups	754	621.23	.824		
	Total	757				
Vigorous Activity Level	Between groups	3	18.78	6.26	7.15	.00
	Within groups	753	659.56	.88		
	Total	756				

#### Gender differences in physical activity level

An independent sample t-test showed a significant difference between gender in physical activity level, *t*(770) = 4.577, *p<*0.01. Males (*M* = 13.41, *SD* = 5.892) engaged in more physical activity than females (*M* = 11.44, *SD* = 5.817).

#### Educational level differences in physical activity

One-way ANOVA showed that there is a significant difference between educational levels in terms of their physical activity level, F (3, 749) = 6.19, *p* <.01 (see Table 3). A Tukey post-hoc test revealed that, participants with primary school level (*M* = 15.22, *SD* = 7.03) engaged significantly more physical activity compared to those of high school level (*M* = 12.45, *SD* = 6.00), Pre-University, form 6 or diploma level (*M* = 11.76, *SD* = 5.81), and degree level and above (*M* = 11.22, *SD* = 5.28).

**Table 3:** One-Way Analysis of Variance of Physical Activity by Educational Level, Marital Status, Occupation, and Age Groups.

Variables	Source	*df*	*SS*	*MS*	*F*	*P*
Educational Level	Between groups	3	638.39	212.80	6.19	.00
	Within groups	749	25744.21	34.37		
	Total	752				
Marital Status	Between groups	4	187.08	46.77	1.34	.25
	Within groups	763	26609.61	34.88		
	Total	767				
Occupation	Between groups	6	524.01	87.34	2.54	.02
	Within groups	752	25864.98	34.40		
	Total	758				
Age Groups	Between groups	6	524.01	87.34	2.54	.02
	Within groups	752	25864.98	34.40		
	Total	758				

#### Age group differences in physical activity

One-way ANOVA showed significant differences between age groups in physical activity, *F* (5,763) = 3.32, *p* <.01 (Table 3). However, Tukey post-hoc test revealed no significant differences among the age groups possibly due to relatively small number of participants in the older age groups.

#### Relationship between physical activity and perceived locus of control

The relationships between perceived locus of control (i.e, internal, powerful others, and chance) and physical activity level (i.e., total physical activity, light, moderate, and vigorous activity) were analysed using Pearson’s correlation. Results showed that there are significant negative correlations between internal perceived locus of control and total physical activity level, *r* = -.095 (*N* = 770), *p* <.01, as well as with vigorous activity, *r* = -.137 (*N* = 768), *p* <.01. This indicates that people with lower internal locus of control engaged more in total physical activity and vigorous activity. Results also showed that there is a very significant negative correlation between powerful-others locus of control and vigorous activity, *r* = -.072 (*N* = 768), *p* <.01. This indicates that people with lower powerful-others locus of control engaged more in vigorous activity.

#### Relationship between physical activity and age

Pearson correlation was conducted to determine the relationship between physical activity level and age. The results showed that there are significant negative relationships between age and total physical activity, *r* = -.130 (*N* = 770), *p* <.01, moderate activity, *r* = -.074 (*N* = 769), *p* <.05, and vigorous activity, *r* = -.146 (*N* = 768), *p* <.01. These results indicate that, as expected, older Malaysians people engage in less total physical activity, moderate activity, and vigorous activity. No significant relationship was found between age and light activity, *r* = .003 (*N* = 768), *p*>.05.

#### Relationship between physical activity and ethnicity

Point-biserial correlations were conducted to determine ethnicity and physical activity level. In terms of Malay, the results showed that there is a significant positive relationships between Malay and total physical activity, *r_pb_* = .071 (*N* = 759), *p* <.05, indicating that compared to other ethnicities, Malays tend to engage more in overall physical activity. No significant relationship was found between Malay and light exercise, *r_pb_ = -*.017 (*N* = 757), *p*>.05, moderate exercise, *r_pb_* = .050 (*N* = 758), *p*>.05, and vigorous exercise, *r_pb_* = .060 (*N* = 757), *p*>.05. In terms of Chinese, there are significant negative relationships between Chinese and total physical activity, *r_pb_* = - .101 (*N* = 759), *p* <.01, light activity, *r_pb_ = -*.088 (*N* = 757), *p* <.01, moderate activity, *r_pb_* = - .101 (*N* = 758), *p* <.01, and vigorous activity, *r_pb_* = .-.078 (*N* = 757), *p* <.05, indicating that Chinese engaged less in all categories of physical activity compared to other ethnicities. Indian participants were more likely than others to engage in light activity, *r_pb_* = .129 (*N* = 757), *p* <.01. But, significant relationships were not found for Indians in relation to total physical activity, *r_pb_* = -.008 (*N* = 759), *p*>.05, moderate activity, *r_pb_* = .055 (*N* = 758), *p*>.05, and vigorous activity, *r_pb_ = -*.045 (*N* = 757), *p*>.05.

#### Relationship between physical activity and occupation

Point-biserial correlations were conducted to examine occupation status and physical activity level. Housewives showed somewhat lower levels of physical activity than other groups for total physical activity, *r_pb_ = -*.090 (*N* = 759), *p* <.05, moderate activity, *r_pb_ = -*.072 (*N* = 758), *p* <.05, and vigorous activity, *r_pb_ = -*.072 (*N* = 757), *p* <.05. No relationship was found between housewife status and light exercise, *r_pb_ = -*.001 (*N* = 757), *p*>.05.

Students also were somewhat less likely to engage vigorous exercise, *r_pb_ = -*.088 (*N* = 757), *p* <.05. No significant relationships were found between student status and total physical activity, *r_pb_ = -*.036 (*N* = 759), *p*>.05, light activity, *r_pb_ = -*.039 (*N* = 757), *p*>.05 and moderate activity, *r_pb_ = -*.007 (*N* = 758), *p*>.05.

The results showed that there are no significant relationships between professional or administrative occupations and total physical activity. Sales/service personnel, however were more likely to engage in vigorous activity, *r_pb_ = -*.079 (*N* = 757), *p* <.05. Those in “other” occupations reported higher total physical activity, *r*_*pb*_ = .105 (*N* = 759), *p* <.01, moderate activity, *r*_*pb*_ = .096 (*N* = 759), *p* <.01, and vigorous activity, *r*_*pb*_ = .*097* (*N* = 759), *p* <.01. No significant result was for "other" occupations and light activity, *r*_*pb*_ = .049 (*N* = 759), *p*>.05.

#### Relationship between physical activity and education

Pearson correlations were conducted to determine the relationship between physical activity level and education. The results showed that there are significant negative relationships between education and total physical activity, *r* = -.135 (*N* = 753), *p* <.01, moderate exercise, *r* = -.111 (*N* = 752), *p* <.01, and vigorous exercise, *r* = -.179 (*N* = 751), *p* <.01. These results indicate that people who have lower education level engage more in overall physical activity, moderate exercise, and vigorous exercise. No significant relationship was found between education and light exercise, *r = -*.014 (*N* = 751), *p*>.05.

#### Multiple regression analysis for perceived locus of control, age, gender, ethnicity, marital status, occupation, and education in predicting total physical activity

To further analyse the effect of internal perceived locus of control, age, gender, ethnicity (i.e., Malay and Chinese), marital status (i.e., married status), occupation (i.e., housewife), and education on total physical activity level, multiple linear regression analyses were conducted. The results (see Table 4) showed that all variables together significantly accounted 8.4% of the variance in predicting total physical activity level, *F* (8, 721) = 8.29, *p* <.01. Specifically, age, gender, Chinese and education showed the strongest negative relationship with total physical activity level. This indicates that males, non-Chinese, younger people, and those with less education engaged more in total physical activity.

**Table 4:** Summary of Multiple Regression Analysis for Perceived Locus of Control, Age, Gender, Ethnicity, Marital Status, Occupation, and Education in Predicting Activity Level.

Variable	*B*	*SE B*	B
Internal locus of control	-.06	.039	-.06
Age	-.07	.022	-.16**
Male	1.61	.453	.13**
Malay	.02	.600	.00
Chinese	-1.44	.574	-.12*
Married	-.04	.570	-.00
Housewife	-1.59	.922	-.07
Education	-1.09	.247	-.17**

*Note*. *R*^*2*^ = .084 (*p* <.01)

*p <.*05*,

**p <.01

#### Multiple regression analysis for ethnicity in predicting light activity

Multiple regression analysis was conducted to determine the effect of Chinese and Indian ethnicity on light exercise. The results showed that Chinese and Indian ethnicity together accounted for 1.8% of the variance in predicting light exercise *F* (2, 754) = 6.968, *p* <.01. However only Indian ethnicity showed a significant relationship with light exercise. Indians were more likely to report light activity.

#### Multiple regression analysis for age, gender, ethnicity, occupation, and education in predicting moderate activity

Multiple regression analysis was conducted to determine the effect of age, male, Chinese, housewife, and education on moderate activity. The results showed that all variables together accounted for 4.4% of the variance in moderate exercise, *F* (5, 725) = 6.74, *p* <.01 with age, Chinese ethnicity and education showing significant negative relationships to moderate activity. Controlling for other variables, Chinese people, those with more education, and older people engaged less in moderate physical activity.

#### Multiple regression analysis for perceived locus of control, age, ethnicity, marital status, occupation and education in predicting vigorous exercise

Multiple regression analysis was conducted to determine the effect of internal and powerful-others locus of control, age, Chinese ethnicity, married status, occupation (i.e., housewife, and student), and education in predicting vigorous activity. The results showed that all variables together significantly accounted 9.2% of the variance in predicting vigorous exercise, *F* (8, 719) = 9.09, *p* <.01 (Table 5). However, only age, Chinese ethnicity, housewife status and higher education levels were significant predictors after controlling for other variables. Older people, ethnic Chinese, housewives, and people with higher levels of education reported lower levels of vigorous activity.

**Table 5:** Summary of Multiple Regression Analysis for Perceived Locus of Control, Age, Ethnicity, Marital Status, Occupation and Education in Predicting Vigorous Exercise.

Variable	*B*	*SE B*	B
Internal locus of control	-.013	.007	-.077
Powerful others locus of control	-.004	.006	-.024
Age	-.013	.003	-.176**
Chinese	-.192	.069	-.101**
Married	-.001	.091	-.001
Housewife	-.382	.142	-.104**
Student	-.091	.104	-.042
Education	-.219	.046	-.216**

*Note*. *R*^*2*^ = .092 (*p* <.01)

**p <.01

## Discussion

Results showed significant differences in physical activity among Malaysians based on ethnicity, gender, education, occupation and age. These results showed no significant difference in physical activity across marital statuses and, when controlling for age and education, locus of control was not significantly related to activity levels.

### Ethnicity

Overall, Chinese participants were the least physically active ethnic group as well as the least likely to report moderate or vigorous activity. This relationship held true even when controlling for all other variables, suggesting, perhaps, that some normative aspects of Malaysian Chinese culture discourage exercise. Indians were most likely to engage in light exercise such as gardening, housework, leisurely walking, bowling, fishing, or carpentry. In keeping with previous findings [19] “Other” races (i.e. not Indian, Chinese, or Malay), were more likely to engage in vigorous activities such as running, heavy yard work, weight training and sports.

### Gender

Malaysian women were less physically active than men. This may relate to traditional, especially Muslim, views of the role of women [13] where physical activity is seen as unfeminine and associated with lower social status. One target for future interventions, thus, may be cultural attitudes among women regarding the importance and desirability of exercise. Similarly, the provision of culturally acceptable venues for women (especially Muslims) to engage in physical activity needs to be considered.

### Marital Status

Contrary to previous studies [25, 26, 27], marital status did not appear to affect the physical activity level of Malaysians. One possible explanation for this is that this was a cross-sectional study whereas two of the previous studies [25, 27] were longitudinal studies more specifically concerned with marital transitions (e.g., from single to married, and from married to divorced). It should also be noted that the current study included an imbalanced distribution of marital statuses (490 singles, 249 married, 14 divorced and 13 widow/widowers).

### Occupation

There were few statistically significant differences in activity scores across seven different occupations. “Other” occupations and sales/service profession were more likely than housewives, students and business owners to be physically active. It is not entirely clear from these results what “other” occupations are. It is, however, likely that many of those in service and “other” professions are engaging in physical activity as part of their job responsibilities (e.g. laborer, cleaner, landscaper, construction etc.). From a cultural standpoint, it is notable that those who presumably have more discretionary time, such as housewives and students, seem to be opting not to use that time to engage in physical activity: Again, this seems to suggest a cultural bias against physical activity as a desirable leisure time pursuit.

### Perceived Locus of Control

Results indicated that people with higher internal locus of control engaged in less physical activity. This result is inconsistent with previous studies and, again, appears to point towards a general cultural orientation in Malaysia which does not prioritize physical activity and, in some cases, casts it in a negative light [32, 33]. Generally, in western countries, people with high internal locus of control tend to engage in more physical activity [34]. For many Malaysians, however, given the choice, they simply do not see being engaged in physical activity as a desirable way to spend their time [35]. This is probably the most important, and perhaps most challenging, issue that needs to be addressed with future interventions at all levels, from individual to societal, if we wish to change physical activity and health trends in Malaysia. Controlling for the effects of education level, and age, the relationship of perceived locus of control on physical activity becomes insignificant. Essentially education and locus of control are interrelated. People who are more educated generally see themselves as having more internal control. The overall trend, however, remains the same; individuals with greater resources seem to be choosing not to engage in physical activity

### Education

From a cross-cultural standpoint, the most noteworthy result of the current study was the relationship of education to physical activity level. Contrary to results from numerous studies conducted in western developed countries [17, 19, 23], educated Malaysians were significantly less likely to engage in all types of physical activity. In fact, when controlling for other variables, education was by far the strongest predictor of whether Malaysians were physically active or not.

Again, this finding seems to indicate an underlying cultural orientation among Malaysians that devalues physical activity. Many Malaysians seem to believe that physical activity is too difficult, not rewarding, uncomfortable, dangerous, or just generally uninteresting [35]. Even secondary school age students, an age which is crucial in forming long-term exercise habits, show a general lack of interest in physical activity [33] which does not bode well for the future. Because of this cultural devaluation, those who have the resources (i.e. education, social class, and locus of control) to engage in healthier, active lifestyles, seem to be choosing not to do so. In this regard, future research should look into some of the following questions: What is the nature of Malaysians early exposure to physical activity (e.g. in neighborhoods, physical education programs, and community recreation programs)? Are there ways to promote physical activity as enjoyable and desirable, especially among youths? Are there accessible and safe environments, such as open space, sidewalks, bike paths, athletic fields, and parks available for interested people to engage in physical activity? Are there ways of promoting even modest levels of activity (e.g. walking vs. driving) as enjoyable, desirable, and beneficial?

### Application of Findings

Evidence shows that physical activity patterns develop relatively early in life [36], but they also are influenced by social and environmental factors [37], as well as attitudes [38]. The findings from this research suggest that rising rates of obesity and non-communicable diseases in Malaysia, at least the portion contributed to by inactivity, relates to a combination of these factors.

Although this study did not look specifically at individual’s reasons for not exercising, other studies [32] as well as less formal surveys of university students and employees have suggested a perceived lack of access to desirable locations such as parks and playing fields, lack of, or poor condition of, sidewalks and bikepaths, as well as safety concerns as barriers to participation in physical activity. This suggests that one priority at various levels of government should be to assure that future development plans include the creation and maintenance of safe and attractive places to engage in physical activity. Also, Malaysia, being in the tropics, is hot all year round, leading many people to organize their activities around shelter, and shade. Developing covered walking tracks, and partially-covered athletic facilities might also encourage activity for some. Zoning and land use regulations that protect recreational spaces also need to be properly enforced in order to keep parks and other resources free from industrial noise, waste, dust, fumes and haze. Local governments also need to be vigilant in assuring that existing sidewalks and bicycle paths are kept clear and well-lit, so that walking and biking remain safe and viable options. Government and employers should also consider incentives for those who choose to commute by walking and cycling.

These findings also point towards societal norms which are more difficult to address. In combination with assuring that appropriate resources are available to all Malaysians who desire to exercise, it will also be important to address underlying attitudes that are apparent in this data. Specifically, those who have greater levels of education and internal locus of control, and those that presumably have more discretionary time, such as housewives and students, seem to be choosing not to be physically active. This points to a fundamental attitude among many Malaysians, especially ethnic Chinese and those with more education; that physical activity is not a priority or not desirable. Future research should look at beliefs about physical activity, why people choose not to be active, and what other activities they are engaging in.

Likely, the best place to start changing such attitudes would be among Malaysia’s youth. Even in our relatively young sample, those who had the resources to be physically active seemed more likely than those from other countries to simply choose not to do so. Research needs to be done on promoting exercise and physical activity as enjoyable ways of spending one’s time. Whether through encouraging parents to get out and play with their young children, or introducing recreational sports during PE classes, or community-based recreation programs, it is important to help children to get in the habit of enjoying themselves through physical activity from an early age. [33].

As far as motivating older, busier segments of the population to exercise, efforts can be made by employers, schools, and the government to educate people as to the benefits of even small amounts of exercise: Using the stairs instead of elevator or escalator, stretching at work, walking or other exercise during lunch breaks, even exercising in front of the TV can all be encouraged through targeted public service announcements and employer-based programs. Government and researchers should work together to design such programs as well as to quantify their results.

## Conclusion

Recent studies show that about 61% of Malaysians are physically inactive [2]. This combined with many unhealthy eating habits has created a society where rates of obesity and non-communicable diseases are increasing at alarming rates. Our findings point to at least one contributor to this problem, which is that Malaysians, in general, seem to devalue the importance or desirability of physical activity as a leisure-time pursuit. Moving into the future it will be important eliminate perceived barriers to participation [32] by ensuring access to safe and appealing locations. Just as importantly, however, at the cultural level, there needs to be more work done to understand the prevailing negative attitudes towards physical activity and to find ways of making physical activity more desirable and interesting for those who have the time and resources to participate.

## Supporting Information

S1 Data This is the data file in SPSS (SAV) format. (SAV)

## References

[1] IPH (Institute for Public Health) (2008) The Third National Health and Morbidity Survey (NHMS III) 2006, General Findings. Ministry of Health Malaysia, Kuala Lumpur.

[2] WHO (2010) WHO Global Info Base: Indicator. Retrieved September 8, 2010, from World Health Organisation: https://apps.who.int/infobase/Indicators.aspx

[3] Rampal L, Rampal S, Khor GL, Zain AM, Ooyub SB, Rahmat RB, et al (2007). A national study on the prevalence of obesity among 16,127 Malaysians. Asia Pacific Journal of Clinical Nutrition, 16, 561–566. 17704038

[4] World Health Organization (2010). Global Recommendations on Physical Activity for Health. Available online at: http://apps.who.int/iris/bitstream/10665/44399/1/9789241599979_eng.pdf 26180873

[5] Ministry of Health Malaysia. (2010) Malaysian Dietary Guidelines. Available online at: http://www.nutriweb.org.my/downloads/Executive%20summary.pdf

[6] Poh BK, Safiah MY, Tahir A, Siti H, Siti NN, Farina Z, Mohd HS (2010). Physical activity pattern and energy expenditure of Malaysian adults: Findings from the Malaysian Adults Nutrition Survey (MANS). Malaysian Journal of Nutrition, 16: 13–37. 22691851

[7] WHO (2003). Diet, nutrition and the prevention of chronic diseases. Report of a Joint WHO/FAO Expert Consultation. WHO Technical Report Series 916. World Health Organization, Geneva 12768890

[8] Popkin BM (2006) Global nutrition dynamics: the world is shifting rapidly toward a diet linked with non-communicable diseases. American Journal of Clinical Nutrition 84: 289–298. 16895874 10.1093/ajcn/84.1.289

[9] Azmi MY, Junidah R, Siti MA, Safiah MY, Fatimah S, Norimah NK, et al (2009). Body mass index of adults: Findings of the Malaysia Adult Nutrition Survey (MANS). Malaysia Journal of Nutrition, 15: 97–119 22691810

[10] Hallal PC, Anderson LB, Bull FC, Guthold R, Haskell W (2012). Global physical activity levels: surveillance progress, pitfalls, and prospects. The Lancet, 380: 247–257. 10.1016/S0140-6736(12)60646-1 22818937

[11] Bauman AE, Reis RS, Sallis JF (2012). Correlates of physical activity: why are some people physically active and others not? The Lancet, 380: 258–271. 10.1016/S0140-6736(12)60735-1 22818938

[12] Tam CL, Bonn G, Yeoh SH, Wong CP (2014). Investigating diet and physical activity in Malaysia: education and physical activity relate to lower levels of physical activity. Frontiers in Psychology. 10.3389/fpsyg.2014.01328 PMC4253661 25520676

[13] Kaur J, Kaur G, Ho BK, Yao WK, Salleh M, Lim KH (2014). Predictors of Physical inactivity among elderly Malaysians: Recommendations for policy planning. Asia-Pacific Journal of Public Health, 27:314–22. 10.1177/1010539513517257 24425796

[14] Razak AM, Fauzee OMS, Latif AR (2010). The perspective of Arabic woman toward sport participation. Journal of Asia Pacific Studies 1: 364–377.

[15] Azevedo MR, Araujo CLP, Reichert FF, Siqueira FV, da Silva MC, Hallal PC (2007). Gender differences in leisure-time physical activity. International Journal of Public Health, 52: 8–15. 17966815 10.1007/s00038-006-5062-1 PMC2778720

[16] Hawkins MS, Storti KL, Richardson CR, King WC, Strath SJ, Holleman RG, et al (2009). Objectively measured physical acivity of USA adults by sex, age, and racial/ethnic groups: A cross-sectional study. International Journal of Behavioral Nutrition and Physical Activity 10.1186/1479-5868-6-31 PMC2701914 19493347

[17] Sherwood NE, Jeffery RW (2000). Implications for Physical Activity Interventions. Annual Review of Nutrition, 20: 21–44. 10940325 10.1146/annurev.nutr.20.1.21

[18] Hallal PC, Victora CG, Wells JC, Lima RC (2003). Physical inactivity: Prevalence and associated variables in Brazilian adults. Medicine and Science in Sports and Exercise 35: 1894–1900. 14600556 10.1249/01.MSS.0000093615.33774.0E

[19] Bauman AE, Sallis JF, Dzewaltowski DA, Owen N (2002). Toward a better understanding of the influences on physical activity: The role of determinants, correlates, causal variables, mediators, moderators, and confounders. American Journal of Preventive Medicine 23,2: 5–14. 12133733 10.1016/s0749-3797(02)00469-5

[20] Teh CH, Lim KK, Chan YY, Lim KH, Azahadi O, Akmar HAH. (2014). The prevalence of physical activity and its associated factors among Malaysian adults: Findings from the National Health and Morbidity Survey 2011. Public Health 128:416–423. 10.1016/j.puhe.2013.10.008 24726414

[21] Ayiesah R (2007). The level of physical activities amongst elderly in a community. *The Journal of Health and Translational Medicine* 10:29–33.

[22] Shaw BA, Spokane LS (2008). Examining the association between education level and physical activity changes during early old age. Journal of Aging and Health 20:767–787 10.1177/0898264308321081 18559963 PMC2570711

[23] Burton NW, Turrel G (2000) Occupation, hours worked, and leisure-time physical activity. Preventive Medicine 31:673–681 11133334 10.1006/pmed.2000.0763

[24] Seiluri T, Lahti J, Rahkonen O, Lahelma E, Lallukka T (2011). Changes in occupational class differences in leisure-time physical activity: A follow-up study. International Journal of Behavioral Nutrition and Physical Activity 10.1186/1479-5868-8-14 PMC3058076 21362168

[25] Engbergm E, Alen M, Kukkonen-Harkula K, Peltonen JE.Tikkanen HO, Pekkarinenm H (2012). Life events and change in leisure time physical activity. Sports Medicine 42: 433–447 10.2165/11597610-000000000-00000 22512413

[26] Pettee KK, Brach JS, Kriska AM, Boudreau R, Richardson CR, Colbert LH, et al (2006) Influence of marital status on physical activity levels among older adults. Medicine and Science in Sports and Exercise, 38: 541–546. 16540843 10.1249/01.mss.0000191346.95244.f7

[27] King AC, Kiernan M, Ahn DK, Wilcox S (1998). The effects of marital transitions on changes in physical activity: Results from a 10-year community study. Annals of Behavioral Medicine 20: 64–69. 9989310 10.1007/BF02884450

[28] Calder BJ, Philips LW, Tybout AM (1982). The concept of external validity. Journal of Consumer Research 9: 240–244.

[29] Yap C.C., Tam C.L., Bonn G. B., Saravanan M & Kadirvel A. (2014). Psychosocial variables influencing diabetes self-management and quality of life In Lumbun G., et al (Eds.) Taylor & Francis Group, London, ISBN 978-1-138-00121-3, pp. 319–325.

[30] Wallston KA, Wallston BS, DeVellis R (1978). Development of the multidimensional health locus of control scales. Health Educ Monogr. 6: 160–170 689890 10.1177/109019817800600107

[31] Godwin M, Streight S, Dyachuk E, van den Hooven EC, Ploemacher J, Seguin R, et al (2008). Simple Lifestyle Indicator Questionnaire (SLIQ). Canadian Family Physician 54: 76–82. 18208960 PMC2293321

[32] DeVries LA (1990). The crisis in Physical Education. Paper presented at 28th International Convention in conjunction with the launching of: PPJM-CAHPER-CIDA PROJECT, 26th-28thNovember, Federal Hotel, Kuala Lumpur.

[33] Wee E (2013). Contemporary issues in the teaching of PE in Malaysia. Journal of Physical Activity, Sports, and Exercise 1: 17–20.

[34] Cobb-Clark DA, Kassenboehmer SC, Schurer S, (2012). Healthy Habits: The connection between diet, exercise, and locus of control. Institute for the study of labor, IZA paper 6789.

[35] Ibrahim S, Karim NA, Oon NL, Ngah WZW (2013). Perceived physical activity barrriers to body weight status and sociodemographic factors among Malaysian men in Klang Valley. BMC Pub Health, 13: 275 23530696 10.1186/1471-2458-13-275 PMC3617120

[36] Dennison BA, Straus JH, Mellitis D, Charney E (1988). “Childhood Physical Fitness Tests: Predictor of Adult Physical Activity Levels?”. Pediatrics 82: 324–330. 3405661

[37] Ding D, Sallis JF, Kerr J, Lee S, Rosenberg DE (2011). Neighborhood environment and physical activity among youth, a review. American Journal of Preventive Medicine 41: 442–55. 21961474 10.1016/j.amepre.2011.06.036

[38] Courneya KS, Conner M, Rhodes RE (2006). Effects of different measurement scales on the variability and predictive validity of the ‘two-component’ model of the theory of planned behavior in the exercise domain. Psychology and Health, 21,5: 557–570.

